# Induction of Gliotoxin Secretion in *Aspergillus fumigatus* by Bacteria-Associated Molecules

**DOI:** 10.1371/journal.pone.0093685

**Published:** 2014-04-04

**Authors:** K. Stefan Svahn, Ulf Göransson, Erja Chryssanthou, Björn Olsen, Jan Sjölin, Adam A. Strömstedt

**Affiliations:** 1 Department of Medicinal Chemistry, Uppsala University, Uppsala, Sweden; 2 Department of Clinical Microbiology, Karolinska University Hospital & Karolinska Institute, Stockholm, Sweden; 3 Department of Medicinal Sciences, Uppsala University, Uppsala, Sweden; Universidade de Sao Paulo, Brazil

## Abstract

*Aspergillus fumigatus* is the most common causative agent of mold diseases in humans, giving rise to life-threatening infections in immunocompromised individuals. One of its secreted metabolites is gliotoxin, a toxic antimicrobial agent. The aim of this study was to determine whether the presence of pathogen-associated molecular patterns in broth cultures of *A. fumigatus* could induce gliotoxin production. Gliotoxin levels were analyzed by ultra-performance liquid chromatography and mass spectrometry. The presence of a bacteria-derived lipopolysaccharide, peptidoglycan, or lipoteichoic acid in the growth media at a concentration of 5 μg/ml increased the gliotoxin concentration in the media by 37%, 65%, and 35%, respectively. The findings reveal a correlation between the concentrations of pathogen-associated molecular patterns and gliotoxin secretion. This shows that there is a yet uncharacterized detection system for such compounds within fungi. Inducing secondary metabolite production by such means in fungi is potentially relevant for drug discovery research. Our results also give a possible explanation for the increased virulence of *A. fumigatus* during bacterial co-infection, one that is important for the transition from colonization to invasiveness in this pulmonary disease.

## Introduction

The discovery of penicillin based on observations made when *Penicillium rubens* and *Staphylococcus aureus* were co-cultured on the same agar plate was arguably the starting point of the Golden Era of Antibiotics [1], [2]. However, *P. rubens* does not provide penicillin in sufficient quantities for mass production under conventional broth conditions. This problem was solved by the discovery of *P. chrysogenum*, which has much higher levels of constitutive penicillin production. Laborious mutation and selection efforts eventually yielded a *P. chrysogenum* strain whose penicillin output exceeded that of Fleming’s mold by a factor of 1000 [3].

To conserve nutrients and avoid self-toxicity as well as resistance development, organisms produce and secrete many antimicrobial agents on a facultative basis. This is especially true for microorganisms such as fungi, which cannot rely as much on nutrient depots and cell-specialization. As a result, fungi would benefit especially from having metabolic pathways that are dormant or downregulated where there are no competing microorganisms. Consequently, it is disputable that fungi secrete the full spectrum of antimicrobial agents in conventional laboratory growth conditions. Methods for activating these pathways could therefore be useful for finding new metabolites or increasing the secretion of known ones, which may be beneficial in drug discovery and production.

Pathogen-associated molecular patterns (PAMPs) are widely used to simulate the presence of bacteria and other pathogens in studies on mammalian immune systems [4], [5]. PAMPs are highly conserved molecular structures, typically essential components of the pathogens cytoplasmic barrier, which can act as general recognition targets for detecting their presence. The detection of PAMPs by the immune system of insects and mammals stimulates innate immune responses and thus prevents infections [6]. Some molecules that have been classified as PAMPs include lipopolysaccharides (LPS) from Gram-negative bacteria, lipoteichoic acid (LTA) from Gram-positive bacteria, and peptidoglycan (PG) associated with both groups of bacteria. These bacterial cell wall and membrane components induce responses in mammalian hosts by binding to Toll-like receptors (TLRs), which are a subfamily of pattern recognition receptors (PRRs). TLRs have been identified in a diverse range of animals but not in fungi [7]–[10]. Although fungi seem to lack TLRs, it is important to know if also fungi have some mechanism for detecting and responding to common bacterial PAMPs.

The fungus *A. fumigatus* is the most common causative agent of mold infections in humans [11]. Pathogenic species of the genus *Aspergillus* often infect severely immunocompromised patients as well as those with more moderate levels of immunosuppression, such as patients with chronic obstructive pulmonary disease (COPD) or critically ill intensive care patients, in whom it can cause serious invasive infections with high mortality rates [11].

The aim of the work reported herein was to determine how three PAMPs (LPS, LTA and PG) affect the secreted metabolite profile of *A. fumigatus.* The use of bacteria or PAMPs to induce fungal metabolite production represents a novel methodology. The traditional way of exploring fungal secondary metabolites is to cultivate them in a standard medium and then analyze the culture filtrate or fungal mycelium [12]. Elicitation using PAMPs could be particularly useful because it is likely that many bioactive fungal metabolites are produced facultatively and so would not be observed under standard cultivation conditions. It was expected that the results obtained in this way might improve our understanding of the pathogenesis of fungal infection, especially the transition from colonization to invasion, and the problems associated with bacterial co-infections [13]. *A. fumigatus* was selected as the object of study because of its potential to provide clinically relevant information as well as new insights into the effects of PAMPs on fungal metabolism.

## Results

**Figure 1:**
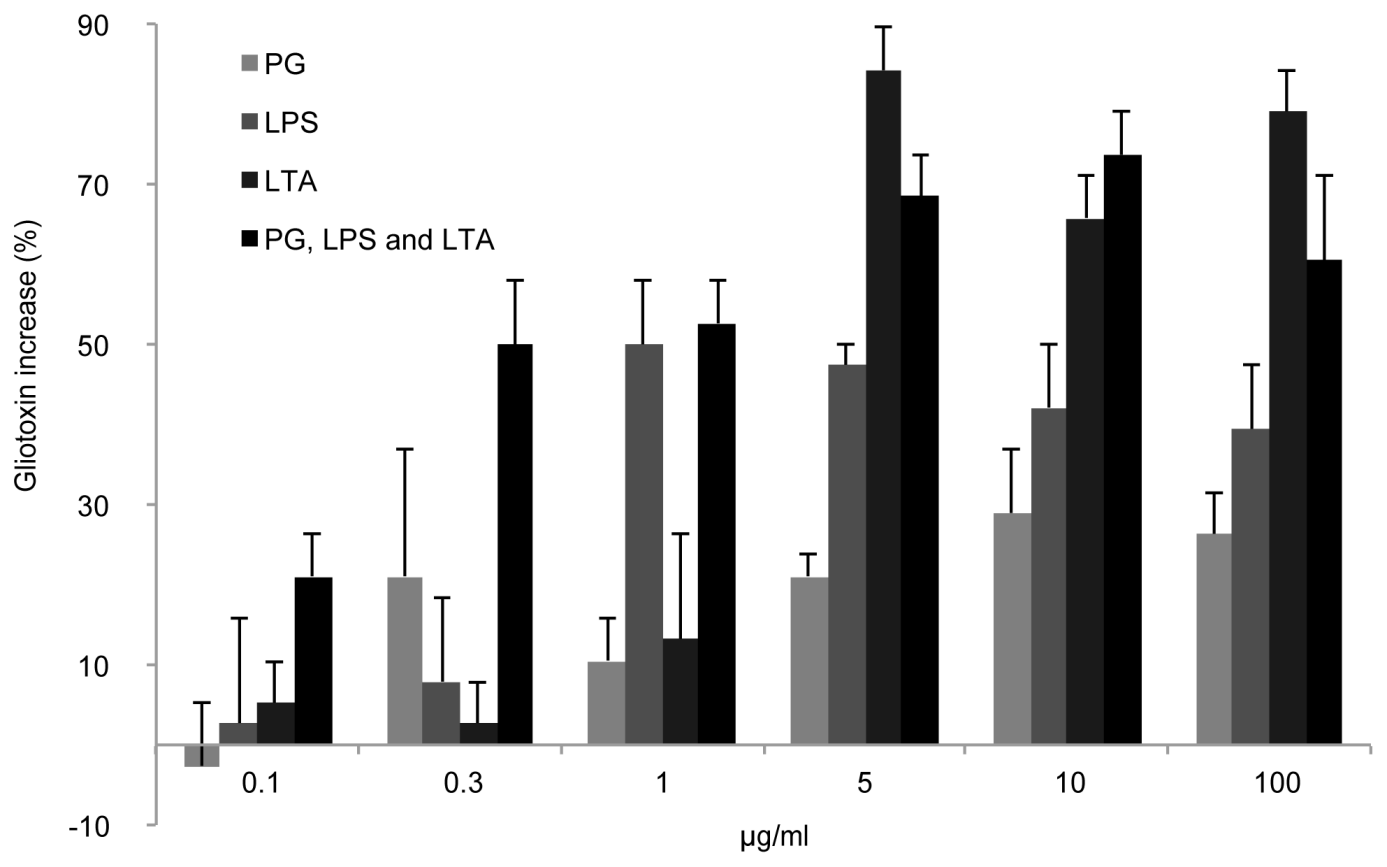
The influence of the PAMPs at concentrations of 0.1–100 μg/ml on the production of gliotoxin by *A. fumigatus,* based on triplicate *A. fumigatus* cultures incubated at 37°C for seven days. The gliotoxin concentrations are normalized against those for the control group (38 μM), and the error bars show the standard error of the reported mean. The gliotoxin level increased in all cultures exposed to PAMPs at concentrations above 0.1 μg/ml.

*A. fumigatus* cultures were grown in the presence of the three selected PAMPs at various concentrations for seven days and the gliotoxin production in each case was compared to that of untreated control cultures. The minimum PAMP concentrations required to cause appreciable increases in gliotoxin secretion were 0.3 μg PG/ml, 1 μg LPS/ml, and 5 μg LTA/ml (Figure 1). The level of gliotoxin release into the media increased with the level of PAMP concentration, up to 10 μg/ml of each PAMP beyond which the gliotoxin increase subsided.

To measure the filtration efficiency, ten 1 ml samples of 1 μM gliotoxin were prepared. Half of the volume of each sample was repeatedly filtered ten times and compared with the unfiltered part of the sample. The efficiency was higher than 99.5% per filtration.

Cultures supplemented with all three PAMPs at a concentration of 0.1 μg PAMPs/ml also exhibited increased gliotoxin secretion relative to control cultures, and a cumulative effect on gliotoxin production was observed for PAMP concentrations below 10 μg/ml. This effect disappeared for combined PAMP concentrations above 10 μg/ml. The results obtained in these preliminary experiments were used to guide the design of the remainder of the study.

**Figure 2:**
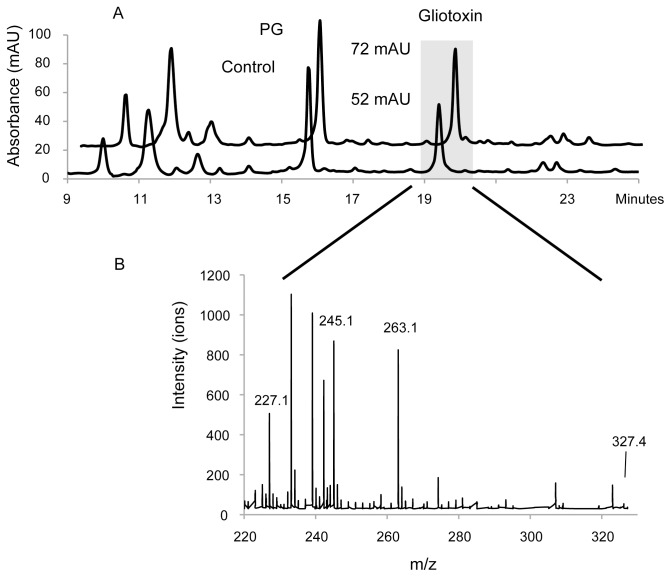
HPLC chromatograms at 254 nm and MS spectra of liquid cultures of *A. fumigatus* after seven days of incubation. **A:** Overlaid chromatograms for the control culture (below) and cultures exposed to 5 μg PG/ml (above). The grey box indicates the gliotoxin peaks. The PG-culture chromatogram is almost identical to that of the control and those for cultures containing LTS and LPS, with the exception of the intensity of the gliotoxin peak at 19.5 minutes. This indicates that the presence of PAMPs did not induce the production of any new metabolites or in any other way radically altered the metabolic profile. **B:** A representative MS spectrum showing the ion pattern of gliotoxin, which features prominent ions at 263.1, 245.1 and 227.1, m/z together with various background ions. The mother ion at 327.4 m/z is also indicated.

After seven days of incubation, the culture broths from the pilot study were analyzed qualitatively using high performance liquid chromatography (HPLC), ultra performance liquid chromatography (UPLC), both reverse phase, and mass spectrometry (MS). The HPLC-derived metabolite profiles of the PAMP-treated cultures showed a marked increase in the gliotoxin peak whiles otherwise strikingly similar to those of the controls (Figure 2). The culture filtrates and mycelial extracts were analyzed in an antimicrobial assay to determine whether they contained any metabolites with antimicrobial activity. The only HPLC fractions of the filtered broths that exhibited antimicrobial activity were those that contained gliotoxin. These gliotoxin-containing fractions inhibited bacterial growth down to a maximum dilution of 1∶64. None of the solid phase extracts of the fungal mycelia showed any antimicrobial activity, and had similar MS-profiles (data not shown). The near-identical metabolite profiles of the treated and untreated cultures and the lack of antimicrobial activity in the gliotoxin-free HPLC fractions of the broths and mycelial extracts indicate that *A. fumigatus* does not produce additional antimicrobial secondary metabolites in the presence of these PAMPs.

**Figure 3:**
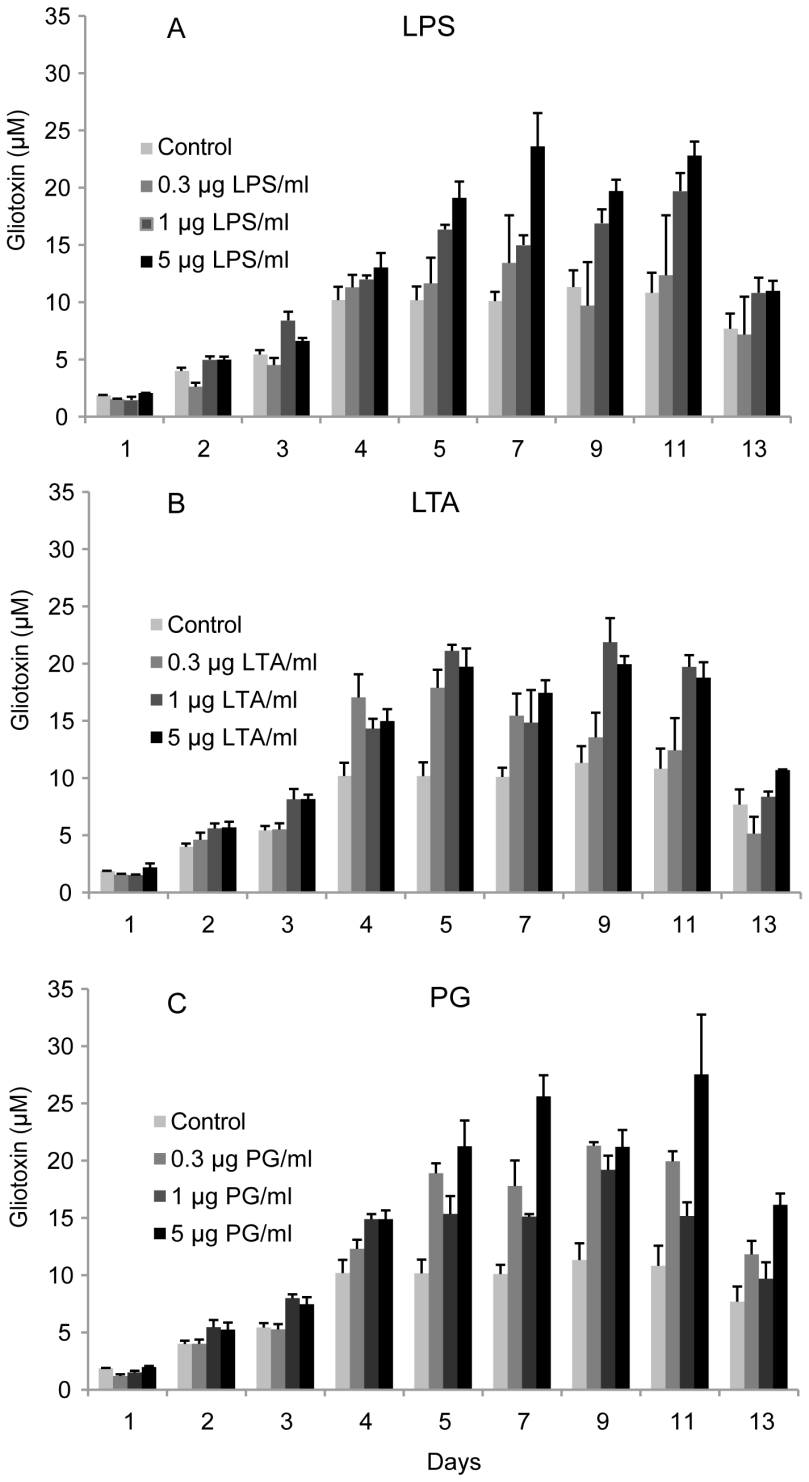
Effects of PAMPs on gliotoxin production by *A. fumigatus* during a 13-day period of incubation at 37°C. The graphs depict gliotoxin concentrations as a function of time for triplicate liquid cultures incubated with LPS (**A**), LTA (**B**) and PG (C) at concentrations of 0.3, 1 or 5 μg/ml. In all experiments, the maximum gliotoxin concentration rose as the PAMP concentration was increased. The highest gliotoxin concentrations were measured between days five and eleven. Error bars show the standard error of the mean for the triplicate experiments.

Over a 13-day period (Figure 3), the differences between the gliotoxin levels produced by PAMP-treated *A. fumigatus* cultures and untreated controls were comparable to those observed in the preliminary 7-day experiments. The gliotoxin concentration was highest between days five and eleven, and tended to increase more rapidly and to peak at a higher level in cultures that were exposed to PAMPs. The addition of LPS, LTA and PG at a concentration of 5 μg per ml of culture medium caused gliotoxin secretion to begin at an earlier stage in the experiment and increased the maximum gliotoxin concentration.

**Figure 4:**
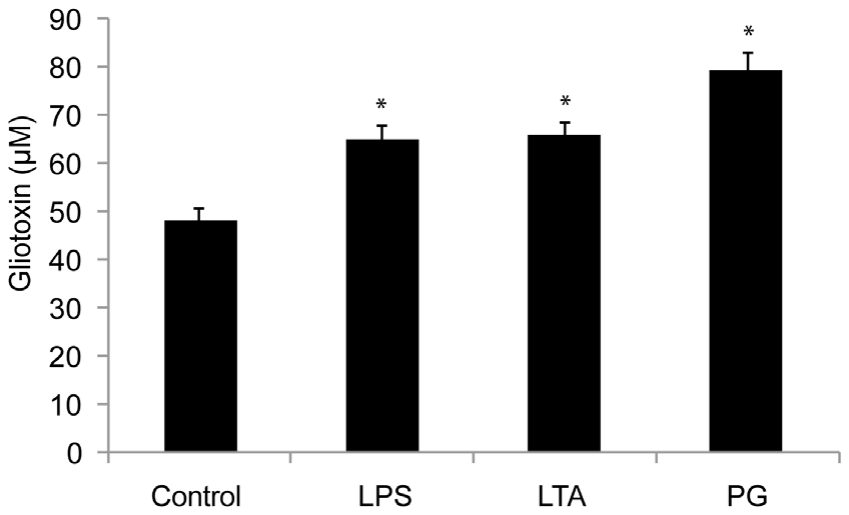
The gliotoxin concentration in the growth media of the four subgroups with 40 cultures each and their standard errors of mean. The Mann-Whitney test indicated that the reference group had a significantly (P<0.001) lower gliotoxin concentration than the other three, indicated by (*), which were grown in the presence of 5 μg of LPS, LTS or PG per ml.

A Mann-Whitney test with a significance threshold of P<0.05 was performed using a batch of 160 cultures that were divided into 4 subgroups of 40 cultures each. The first three groups were cultured in media containing 5 μg/ml of LPS, LTA or PG, respectively, while the fourth served as an untreated control. There were significant differences (P<0.001) between all of the PAMP-treated cultures and the control group: the concentration of gliotoxin in the control was 48.1 μM while the gliotoxin concentrations for the LPS, LTA, and PG cultures were 64.9 μM (+35%), 65.8 μM (+37%) or 79.2 μM (+65%), respectively (Figure 4).

## Discussion

The addition of PAMPs to liquid cultures of *A. fumigatus* increased their secretion of gliotoxin by up to 65% after seven days of incubation. This demonstrates that there is a correlation between the presence of PAMPs and the secretion of gliotoxin. Gliotoxin is an epipolythiodioxopiperazin and a secondary metabolite from *A. fumigatus* with a characteristic disulfide bridge which is essential for its activity [14], [15]. Gliotoxin is a product of the *gli* non-ribosomal peptide synthetase gene cluster and its biosynthesis regulated by transcript factors from *gliZ*, *gliI*, and *gliP* [16]. This study, however, does not differentiate between secretion and regulation of biosynthesis.

The available data on the *A. fumigatus* genome have recently been reviewed, showing that most of its receptors have yet to be characterized [17]. Based on our findings, it is possible that one or more of these uncharacterized receptors may be sensitive to PAMPs. Furthermore, little is known about the interaction between fungi and bacteria. [18] [19] [20] Even less is known of the underlying factors of elicitation in fungi, and the effects of PAMPs on fungal metabolism and growth have not yet been described [21], [22]. While there may conceivably be several receptors that are involved in these PAMP recognition pathways, our results suggest that they are governed by a mutual feedback system or exhibit some degree of mutual saturation in signal transduction because the additive effect of different PAMPs is weak and not discernible at higher concentrations.

PRRs in plants and mammals detect microbial pathogens, causing the activation of host defense systems and immunomodulatory responses. Many microbe-associated molecular patterns exist, but their interactions with plant PRRs have yet to be characterized [21]. For mammals, in general, the TLR4 receptor binds to the lipid A moiety of LPS [23] while the TLR2 receptor detects PG and LTA [24], [25]. However, fungi-associated molecular patterns such as zymosan, β-glycan and mannan are also detected via TLR2 and TLR4 [26]. If *A. fumigatus* does express proteins analogous to the TLR-like receptors, these must somehow be able to distinguish between their own recognition substances and those of competing organisms. Although no known PAMP-receptor like proteins have yet been recognized in fungal genomes [7]–[10], our results indicate that at least in the case of *A. fumigatus*, there are PAMP detection systems whose activation can increase the secretion of specific metabolites – in our case gliotoxin.

Although there are examples of new compounds identified with help of bacterial and fungal co-cultivation [27], there has been no previous study on the effect of individual PAMPs on fungal metabolism. The ubiquitous bacterial molecules used as PAMPs in this study could possibly be universal stimuli for the production of antimicrobial agents by most organisms. While the traditional approach to drug discovery does not use any such stimulation, it works well for identifying constitutively expressed antimicrobial agents. However, such constitutive expression is costly for the host in terms of nutrient conservation, self-toxicity and resistance development. Multicellular organisms with high levels of cellular specialization, such as mammals, have evolved adaptations to circumvent these fitness costs, including nutrient depots, sacrificial cells and redundancy in their immune responses. Even so, the production and secretion of antimicrobial agents during mammalian immune responses generally occurs on a facultative basis. This is well exemplified by the prevalence of situation-dependent production of antimicrobial peptides in the human gut [28]. We reason that fungi, which exhibit a lower degree of multicellular complexity than mammals, should be more sensitive to the fitness costs associated with superfluous antimicrobial production, and thus even more inclined toward facultative production. An unfortunate implication of this line of reasoning is that when fungi and other organisms pass through typical antibiotic screening programs, much of their arsenal is likely to remain un-induced and undetected. The results presented herein indicate the existence of one or several PAMP-recognition systems in *A. fumigatus*, although these systems are not yet characterized. Furthermore, we show that these PAMPs have significant and specific effect on the release of an antimicrobial secondary metabolite. This highlights the need for a re-evaluation of traditional approaches to fungal antibiotic screening.

Molds of the genus *Aspergillus* are ubiquitous in nature, growing in soils, on plants and on organic debris. They are common in both outdoor and indoor air, including that of hospitals as well as in water, food items and dust [29]. People are therefore widely exposed to airborne *Aspergillus* spp. conidia and at risk of colonization, primarily in the respiratory tract. Normally, fungi that manage to penetrate the respiratory mucosa are destroyed quickly by innate immune cells. *Aspergillus* spp. infections are therefore exceedingly rare in immunocompetent individuals. However, patients with conditions that cause immunodeficiency (e.g. HIV) or who are undergoing immunosuppressive treatments (e.g. chemotherapy) are at a high risk of contracting invasive aspergillosis [11], especially if the polymorphonuclear neutrophils (PMNs) and T-cells are affected.

The effector cells of the innate immune system play a crucial role in the transition from early colonization to invasive aspergillosis. Alveolar macrophages can rapidly phagocytose and destroy conidia, while PMNs prevent the germination of the conidia and their subsequent transition to the hyphal form, which is a key prerequisite for invasion [30], [31]. A number of antimicrobial molecules secreted by the respiratory epithelium also seem to be important in the elimination of conidia [29]. While the condition of the host immune response is the most decisive factor in the development of aspergillosis, the results presented herein demonstrate that the presence of bacteria-associated molecules can increase the fungal toxin production and thereby increase its virulence.

Gliotoxin has been shown to induce apoptosis and inhibit multiple processes associated with the activation, differentiation and function of macrophages, PMNs and T-cells [32]–[34]. After inhalation of spores, germination leads to gliotoxin secretion. This secretion is further stimulated by the presence of surrounding bacteria and may thus depress the conidia elimination process and shift the balance in favor of invasion. In addition, gliotoxin-induced apoptosis of lung epithelial cells may breach this physical barrier that protects the underlying tissues and thereby facilitating invasion [33]. Patients suffering from COPD, for example, are frequently colonized by large numbers of Gram-negative or Gram-positive bacteria, which produce and release large quantities of LPS, PG and LTA. Although the PAMP concentration required to elicit increased gliotoxin production in our experiments might be high as compared to typical systemic concentrations of bacteria infected patients, the local concentrations of PAMPs on the infected mucosal surfaces of the lung is likely to be much higher. In summary, we propose a theory that would explain how a bacterial infection possibly aggravates the mold co-infection rather than the traditional view that the mold infection clears a path for subsequent bacterial co-infections. In support of this, a recent study of aspergillosis patients showed that a systemic antibacterial treatment targeting the frequent co-infecting *Pseudomonas aeruginosa*, had a side effect of significantly diminishing the presence of *Asperigillus fumigatus* in the lungs [13]. These results therefore advocate further investigation of the need to prioritize preventive suppression of pulmonary bacterial colonization in patients that have a high risk of contracting aspergillosis.

## Materials and Methods

### Cultivation and Antimicrobial Assays

*Aspergillus fumigatus* ATCC204305 was cultured on potato-dextrose slopes (BD, USA) for seven days at 37°C. The conidia were harvested and suspended in RPMI 1640 broth (Sigma-Aldrich, St. Louis, MO) and the conidial count was adjusted to a concentration of 2–6×10^6^ conidia/ml. The conidial suspension was then divided into separate 10 ml cultures in 15 ml Falcon tubes. Every culture to be used in a given experiment was prepared using an inoculation from the same conidial suspension batch and incubated at 37°C under static and aerobic conditions.

To begin with, the PAMP concentrations required to induce discernible changes in gliotoxin secretion by *A. fumigatus* were determined. Cultures to be grown in the presence of one PAMP were treated with 300 μl of a dimethyl sulfoxide (DMSO) containing LPS, PG and/or LTA at concentrations of 0.1–100 μg/ml. Control cultures were treated with 300 μl of pure DMSO. All PAMPs were obtained from Sigma-Aldrich and isolated from *Escherichia coli* (LPS) or *Staphylococcus aureus* (LTA and PG).

These cultures were incubated for seven days and analyzed by HPLC, UPLC, and MS. The control group and cultures mixed with 5 μg/ml of all three PAMPs were fractionated by HPLC and used in antimicrobial assays to determine whether they secreted any antimicrobial metabolites other than gliotoxin. The mycelial growth was extracted three times with methylene chloride (using three volumes of solvent to one volume of mycelium), for two hours each, after which the whole extraction procedure was repeated using methanol as the solvent. The combined extracts were then evaporated, re-dissolved in 10 ml RPMI, and used in antimicrobial assays, and MS analysis. Antimicrobial assays were conducted using four bacterial strains: *Escherichia coli* ATCC 25922, *Staphylococcus aureus* ATCC 29213, *Pseudomonas aeruginosa* ATCC 27853, and the yeast *Candida albicans* ATCC 90028. The assay procedure is described elsewhere [35]. Both the extracts of the *A. fumigatus* mycelial growths and the culture filtrates were tested at dilutions ranging from 1∶1 to 1∶128.

Throughout the study, the measured gliotoxin concentrations differed between different groups of cultures grown under ostensibly identical conditions. For example, figure 3C shows an average gliotoxin concentration of 26 μM for one group of cultures grown in media containing 5 μg PG/ml whereas figure 4 shows that a different set of cultures grown under these conditions had an average gliotoxin concentration of 79 μM. This difference was due to the culture preparation procedure: when *A. fumigatus* conidia were added to the culture media during the preparation of different culture batches, initial conidial concentrations of between 2 and 6 million conidia per ml were considered acceptable. Consequently, different culture batches could potentially have had a threefold difference in starting concentrations of fungal conidia. However, the study was designed so that all of the cultures that were compared in the Mann-Whitney test originated from the same fungal batch and so all cultures within this batch had the same conidial concentration. The same approach was used to generate the data shown in figures 1, 2 and 3.

### Chemical Analysis

The metabolite profile of the culture broth was analyzed over a period of 13 days. Triplicate cultures were incubated in the presence of 0.3, 1.0, or 5.0 μg/ml of LPS, LTA or PG. Aliquots of 100 μl were taken from each culture on a daily basis during the first five days of growth and then on every other day over the subsequent 13-day period.

All chemicals used were of analytical grade or better. All culture samples were filtered using 0.2 μm filter centrifuge tubes (National Scientific, Rockwood, TN) before liquid chromatography analysis. Prior to analysis by MS, the filtered samples were diluted to 1∶50 in a 10% solution of aqueous acetonitrile containing 0.1% (v/v) formic acid and analyzed using a Nano Acquity UPLC system coupled to a quadropole time-of-flight mass spectrometer (Qtof Micro, Waters, Milford, MA). The concentration of gliotoxin was determined by focusing on the ion at 245.1 m/z. External standard curves were created using gliotoxin (Sigma-Aldrich, St. Louis, MO). The UPLC was equipped with a 75 μm×150 mm, 1.7 μm C18 column that was eluted at a flow rate of 0.250 μl/min using a gradient of 2–90% acetonitrile in water with 0.1% (v/v) formic acid over 49 minutes. The mass spectrometer was calibrated using [Glu^1^]-Fibrinopeptide B human to a mass accuracy of more than 5 ppm, at a capillary voltage of 4300 V and a cone voltage of 5 V. All spectra and chromatograms were analyzed using the MassLynx version 4.0 software package (Waters, Milford, MA,). HPLC analysis was carried out on an Äkta Basic 10 HPLC system (Amersham Pharmacia Biotech, Sweden) with an Ace 250×4.6 mm C18 column (Ace, Aberdeen, UK) packed with 5 μm particles. The flow rate was set to 1 ml/min and the UV-900 Detector was operated at wavelengths of 280, 254 and 215 nm. The mobile phases consisted of 10% (A) and 60% (B) acetonitrile respectively, both containing 0.05% trifluoroacetic acid. The gradient was set to 10–100% (B) over 21 minutes.

### Statistics

A Mann-Whitney test was used in preference to a t-test when evaluating the differences in the gliotoxin concentrations between cultures with and without PAMPs because the gliotoxin concentration was not always normally distributed under these incubation conditions. All data were analyzed using version 2.25.2 of the R studio software package (Boston, MA, USA).
